# Abundance and diversity of zooplankton in semi intensive prawn (*Macrobrachium rosenbergii*) farm

**DOI:** 10.1186/2193-1801-2-183

**Published:** 2013-04-24

**Authors:** Jadobendro Shil, Alokesh Kumar Ghosh, S M Bazlur Rahaman

**Affiliations:** Fisheries and Marine Resource Technology Discipline, Life Science School, Khulna University, Khulna, 9208 Bangladesh

**Keywords:** Zooplankton, Diversity, Abundance, Copepod

## Abstract

The present study was carried out on the seasonal abundance and diversity of zooplankton in a semi- intensive prawn farm of Bagerhat district from July to December, 2008. Plankton samples were collected by conical shaped monofilament nylon net (Plankton net) and Lugol’s solution was used for preservation. The zooplankton abundance was influenced by physico-chemical factors. During the study period 11 genera of zooplankton under 5 orders were recorded from the study ponds namely Copepoda, Rotifera, Cladocera, Ostracoda and Crustacean Larvae. Among all groups copepod was the dominant order. The percentages of Copepoda, Rotifera, Cladocera, Ostracoda and Crustacean Larvae in semi-intensive culture system were 54%, 28%, 12%, 4% and 2% respectively. But the genera *Brachionus* under the order of Rotifer was dominant among all other genera. *Cyclops* and *Helidiaptomus* under the order of Copepod were the 2nd dominant genera. Numbers of zooplankton species were recorded to be the highest in summer season and minimum at early winter season. Highest number of zooplankton found at the month of October. Total zooplankton shows significant positive relationship with water temperature ((r = +0.384), Dissolve Oxygen(r = +0.113), pH(r = +0.320), Free CO2 (r = +0.319), Alkalinity(r = +0.269), Hardness (r = +0.402) and negative relationship with Salinity(r = -0.486), Transparency(r = -0.693). The findings of the present study will help to improve the management strategies of shrimp culture system.

## Introduction

Fish and Fisheries play an important role in the social and economic life of Bangladesh in terms of income, nutrition, employment and foreign exchange earnings. The people of Bangladesh depend on fish as the principal source of animal protein. It contributes around 3.74% to the GDP and 4.04% to foreign exchange earnings through export. Fish provide 58% of national animal protein consumption. Fisheries provide livelihood to about 12 million people of the country directly and indirectly (Department of Fisheries, 2009). Among fisheries products, prawn (*Macrobrachium rosenbergii*) and shrimp (*Penaeus monodon*) are very important. In world market, prawn is very attractive product. Its demand knows no bounds for its nutrition and taste. For increasing the demand of prawn in international market prawn culture has been increasing from the 70th decades and now it is known as a large commerce. This commerce has a great contribution on the increase of national income, industrialization, employment and earning of foreign exchange. Greater Khulna region is consider as the most suitable area for prawn cultivation as over 75% of prawn production comes from this coastal area. Gher farming is now primary livelihood strategy of more than 100,000 rural households in the south west region of Bangladesh. The successfully farm management depends on natural feed and good water quality management into culturable pond. Plankton is microscopic organisms that formulate the base of food chains and food webs in all aquatic ecosystems. Zooplankton feed on Phytoplankton and directly related with the growth of fish especially prawn and shrimp. So the study of zooplankton is so much important. Most forms of zooplankton are motile, and thus their distribution both vertically and horizontally may be quite variable. Zooplankton plays an important food item of omnivorous and carnivorous fishes (Alam *et al.,*^1987^). The larvae of carps feed mostly on zooplankton (Bardach *et al.,*^1972^), because zooplankton provide the necessary amount of protein requires for the rapid growth and development of different organs specially the gonad of fishes. The zooplankton contributes about 23% of the food item of shrimp (Alam *et al.,*^1987^, 32% of the *Notopterus notopterus* (Mustafa and Ahmed, 1979), 47% of the *Catla catla* and 6.37% of the *Labeo rohita* (Menon *et al.,*^1981^). The larvae of fish especially shrimp mostly feed on zooplankton because zooplankton provide the necessary amount of protein requires for the rapid growth of the shrimp (Bardach *et al.,*^1972^). The abundance and diversity of plankton also affect the survival and growth rate of cultured fish. Their abundance and diversity greatly influence the culture system through maintaining oxygen concentration in water, ensuring the balance between O_2_ and CO_2_, enhancing the decomposition of organic matters accumulated in the pond, preventing the development of demersal microalgae and pests, stabilizing water temperature in the pond, regulating pH value and the ecosystem of the pond and also minimizing the variation of water quality parameters (Das and Bhuyan 1974). The relationship between the physico-chemical parameters and plankton production of pond water and their relation with monthly fluctuations of zooplankton are of great importance and basically very much essential in case of fish culture and fisheries management. Fishes are more dependent on water temperature, pH, dissolve oxygen, free CO_2,_ alkalinity and some other salts for growth and developments (Nikolsky, 1963). Any changes of these parameters may affect the growth; development and maturity of fish (Nikolsky, 1963 and Jhingran, 1985).

In order to fisheries development and to increase the present production level, proper and scientific management is essential in which the knowledge of water quality and natural productivity plays an important role. For broader economic objectives, identification, estimation of plankton abundance is essential for proper exploitation of aquatic resources that leads to economic benefits, employment and balance of ecosystem. In the light of the above, the present study has been undertaken to know about zooplankton abundance and water quality parameters in the semi-intensive prawn culture system.

## Materials and methods

### Sampling station and sampling design

**Figure 1:**
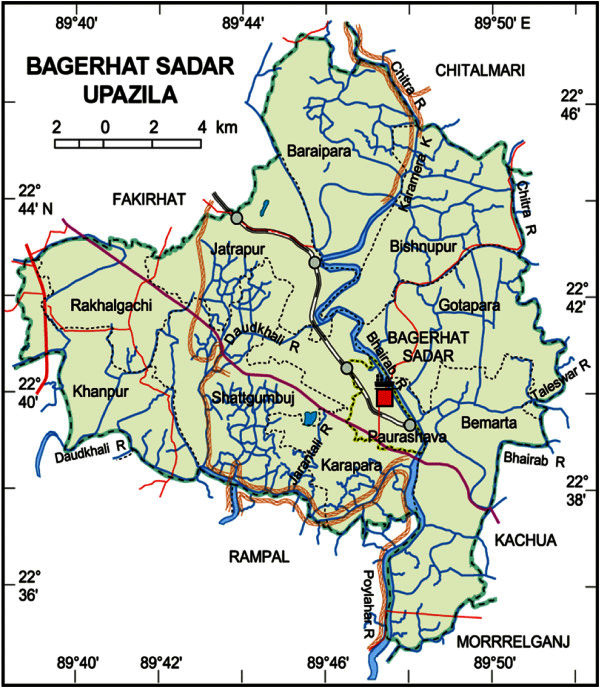
**Map of Bagerhat sadar.**

A semi-intensive prawn farm was selected for the present study which was situated beside the Bagerhat town in Bagerhat district and geographically located at 22°36′ to 22°46′ north latitude and 89°40′ to 89°50′ east longitude (Figure 1). The farm was on the Bhairab river of about 70 decimal and a depth 5-6 ft. The pond was joined with a canal so that fresh water could enter the pond. Two ponds were selected where semi-intensive culture was practiced. The samples were collected at 30 days interval from June 12th to December 10th, 2008. Three representative samples were collected to increase accuracy of the result.

### Plankton collection and preservation

Plankton samples were collected in monthly intervals at 10.30 am on each sampling date by conical shaped monofilament nylon net (Plankton net). The mesh size of the plankton net was 90 μm and the diameter of the net at mouth was 30 cm. Samples were collected from pelagic waters of the ponds from different parts of the ponds. The water was passed down through the net and the plankton condensed at the lower end of the plankton net then it was collected into a glass test tube and fixed firmly (Welch, 1948). After collection, the plankton materials were transferred into glass bottles and preserved with Lugol’s solution. About 250 ml samples of plankton were preserved with 1.5 ml Lugol’s solution. After preservation the plankton samples were carried out to the Biology laboratory of Fisheries and Marine Resource Technology Discipline for further analyses.

### Plankton identification

Plankton cells were enumerated under a light microscope by using Sedgwick-Rafter cell. Recognition of species is a matter of experience. Thus, a series of pencil and ink drawing on postcards of the species of the observed were prepared to identify the organisms. Identification was done following Davis (1956), Moniruzzaman (1997), Zheng (1984), Todd (1991) and Charles (1955).

### Counting

The quantitative enumeration of the zooplankton was carried out with the help of a Sedgwick-Rafter (S-R) counting cell which is 50 mm long, 20 mm wide and 1 mm deep. Before filling the S-R cell with sample, the cover glasses were diagonally placed across the cell and then samples were transferred with a large bore pipette so that no air bubbles in the cell covers were formed. The S-R cell was let stunned for at least 15 minutes to settle zooplankton. Then plankton on the bottom of the S-R cell was enumerated by compound microscope. By moving the mechanical stage, the entire bottom of the slide area was examined carefully. To achieve a random sampling, each time 3 fields were examined for each sample and an average of the counts had been recorded. The organisms thus counted, were expressed as cells per liter (cells) of the sample. From each sample 20 cells counts in 3 slides have been made to achieve random counts and an average of the counts has been recorded. Number of plankton (Zooplankton) in the S-R cell was derived from the following formula.

Where, C = Number of Organisms Counted; L = length of each strip (S-R cell length) in mm; D = depth of a strip (whipple grid image width) in mm; W = width of each stripe (mm); S = number of strips counted. The number of cells per mm was multiplied by a correction factor to adjust the number of organisms per liter (APHA, 1976).

### Measurement of physicochemical parameters

The physicochemical parameters such as temperature, transparency, pH, free carbon dioxide (CO_2_), dissolve oxygen (DO), alkalinity, hardness and salinity were measured during the study period.

### Statistical analyses

Correlation and regression between various water quality parameters and abundance were done using Microsoft Excel Program and SPSS program.

## Results

### Diversity of zooplankton

A checklist of zooplankton occurred in semi-intensive culture system is shown in Table 1. The identified zooplankton populations were five orders namely, Copepoda, Rotifera, Cladocera, Ostracoda and Different Crustacean Larvae. A total of 11 zooplankton genera under the 5 orders were recorded from the study ponds. Among the collected zooplankton, the order Copepoda was dominant with 4 genus followed by Rotifera (2 genus), Cladocera (2 genus) Ostracoda (2 genus) and shrimp larvae (1 genus) also found. *Brachionus and Filinia* was belonging to Rotifera. *Brachionus* was the dominant genus while the genus *Filinia* was observed only in few months. *Diaptomus, Helidiaptomus, Cyclops and Mesocyclops* was under the order of Copepoda of which *Cyclops* was dominant. *Diphansoma and Daphnia* was observed belong to order of Cladocera and *Daphnia* was dominant*.* Cypris and *Cyclocypris* were found under the order of Ostracoda both of the genera were found only for few months. Crustacean larvae was found every month but it was not dominant.

**Table 1:** **Species composition of zooplankton in semi-intensive culture systems**

Sampling time and date	Class/Order	Genus name of Zooplankton
July (06/07/2008)	Copepoda	*Cyclops sp., Mesocyclops sp.,Diaptomus sp., Helidiaptomus sp.*
	Rotifera	*Brachionus sp., Filinia sp.*
	Cladocera	*Diphansoma sp., Daphniasp.*
	Ostracoda	
	Crustacean larvae	*Shrimp larvae*
August (07/08/2008)	Copepoda	*Cyclops sp., Mesocyclops sp. Diaptomus sp.,Helidiaptomus sp*
	Rotifera	*Brachionus . Filnia .*
	Cladocera	*Diphansoma, Daphnia*
	Ostracoda	*Cypris*
	Crustacean larvae	*Shrimp larvae*
September (08/09/2008)	Copepoda	*Cyclops sp., Mesocyclops sp. Diaptomus sp., Helidiaptomus sp.*
	Rotifera	*Brachionus sp.*
	Cladocera	*Diphansoma sp, Daphnia sp.*
	Ostracoda	
	Crustacean larvae	*Shrimp larvae*
October (06/10/2008)	Copepoda	*Cyclops sp., Mesocyclops sp. Diaptomus sp., Helidiaptomus sp.*
	Rotifera	*Brachionus sp. Filnia sp*
	Cladocera	*Diphansoma sp.*
	Ostracoda	*- cyclocypris cypris*
	Crustacean larvae	Shrimp larvae
November (08/11/2008)	Copepoda	*Cyclops sp., Mesocyclops sp. Diaptomus sp., Helidiaptomus sp.*
	Rotifera	*Brachionus,Filinia sp*.
	Cladocera	*Daphnia sp.*
	Ostracoda	*Cyclocypris sp. cypris*
	Crustacean larvae	*Shrimp larvae*
December (07/12/2008)	Copepoda	*Cyclops sp., Mesocyclops sp., Helidiaptomus sp.*
	Rotifera	*Brachionus sp. ,Filnia sp*
	Cladocera	*Diphansoma sp., Daphniasp*
	Ostracoda	*cypris*
	Crustacean larvae	*Shrimp larvae*

### Abundance of zooplankton

Abundance of zooplankton in semi-intensive culture systems is shown in Table 2. The maximum zooplankton abundance (1570 individuals/L) was recorded in the month of October and the minimum abundance (1102 individuals/L)) was noticed in the month of September.

**Table 2:** **Abundance of zooplankton (individual/L) during study period**

Organisms	July	August	September	October	November	December	Total
**Rotifera**							
*Brachionus*	220	110	432	320	375	475	1932
*Filinia*	20	30	0	0	70	80	200
**Copepoda**							
*Diaptomus*	120	60	60	180	20	0	440
*Helidiaptomus*	140	120	160	650	150	40	1260
*Cyclops*	430	450	210	170	120	100	1480
*Mesocyclops*	325	245	60	60	120	140	950
**Cladocera**							0
*Diphansoma*	30	90	50	70	0	40	280
*Daphnia*	120	145	120	0	80	170	635
**Ostracoda**							0
*cyclocypris*	0	0	0	50	20	0	70
*cypris*	0	45	0	60	60	60	225
**Crustacean Larvae**							0
*Nauplius larva*	30	40	10	10	20	30	140
**Total**	1435	1335	1102	1570	1035	1135	7612

**Figure 2:**
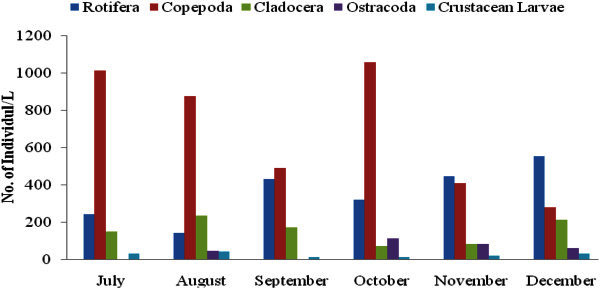
**Different groups of zooplankton during study period.**

The different orders of zooplankton which were noticed in different month are shown by Figure 2. Among different months, Copepoda (1115 individuals/L) was highest in the month of July, Rotifera (555 individuals/L) was highest in the month of December, Cladocera (235 individuals/L)) was highest in the month of August, Ostracoda (110 individuals/L) was highest in the month of October and crustacean (40 individuals/L) was highest in the month of August.

### Water quality parameters

A total of 8 water quality parameters were measured while collecting plankton samples. Their maximum, minimum, mean and standard deviation values are given in Table 3.

**Table 3:** **Some water quality parameters during the study period**

Parameters	Maximum	Minimum	Mean ± SD
Water tem.(oc)	30	22	27.5 ± 2.880972058
Transparency	26	18	22.66667 ± 2.73252
pH	8.1	7.1	7.55 ± 0.3937
Free CO_2_	9.94	6.7	8.24 ± 1.165161
Alkalinity	233.3	116.24	163.26 ± 51.46184
Hardness	1623.33	1022.54	1222.898 ± 222.2622
DO	6	3	4.61 ± 1.20025
Salinity	4.5	2	3.25 ± 1.004

### Correlations of zooplankton abundance with different water quality parameters

Zooplankton abundance was positively correlated with pH, Dissolve oxygen and temperature while negative correlations were found with transparency and salinity in semi-intensive culture system. Correlations of zooplankton abundance with water quality parameters in semi intensive culture are given by Table 4.

**Table 4:** **The co-efficient of correlation of total zooplankton and physicochemical parameters**

Sl. No.	Particulars	Co-efficient of correlation	Comments
1.	Total Zooplankton Vs Water Temperature	.384	Significant
2.	Total Zooplankton Vs Transparency	-.693	Inversely related
3.	Total Zooplankton Vs pH	.320	Significant
4.	Total Zooplankton Vs Free CO2	.319	Significant
5.	Total Zooplankton Vs DO	.113	Significant
6.	Total Zooplankton Vs Alkalinity	.269	Significant
7.	Total Zooplankton Vs Salinity	-.486	Inversely related
8**.**	Total Zooplankton Vs Hardness	.402	Significant

### Regression of zooplankton abundance with different water quality parameters

**Figure 3:**
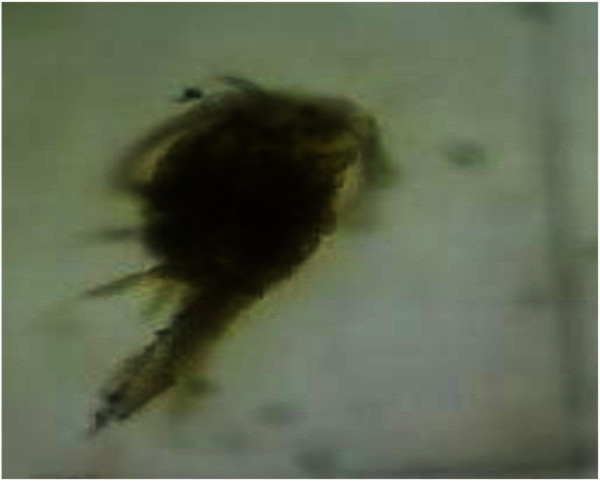
***Brachionus*sp.**

**Figure 4:**
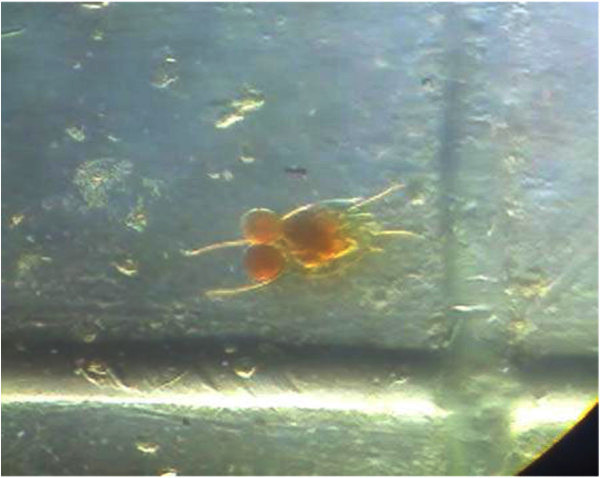
***Filnia.***

**Figure 5:**
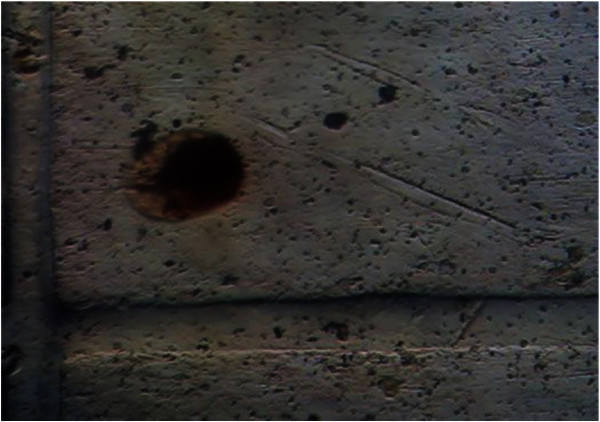
***Cypris.***

**Figure 6:**
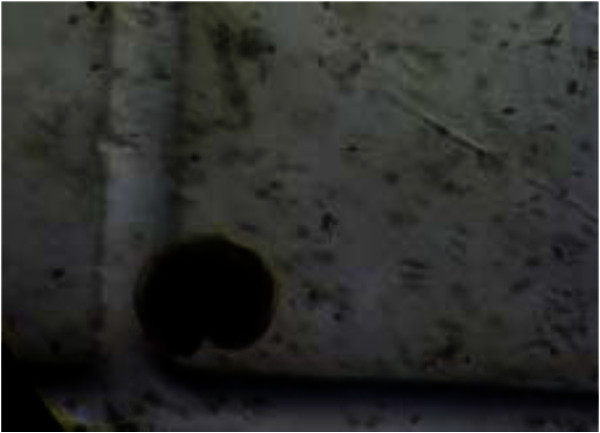
***Cyclocypris.***

**Figure 7:**
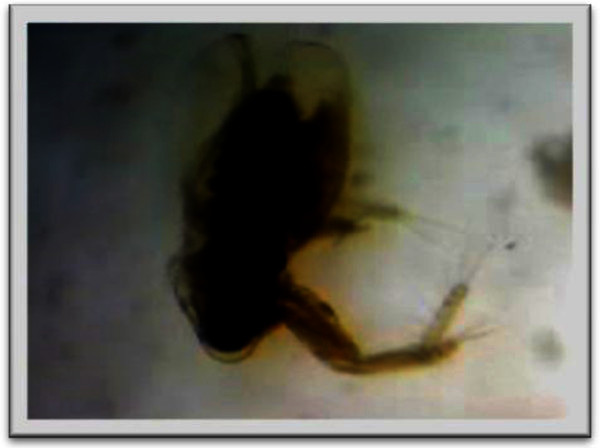
***Diphansoma.***

**Figure 8:**
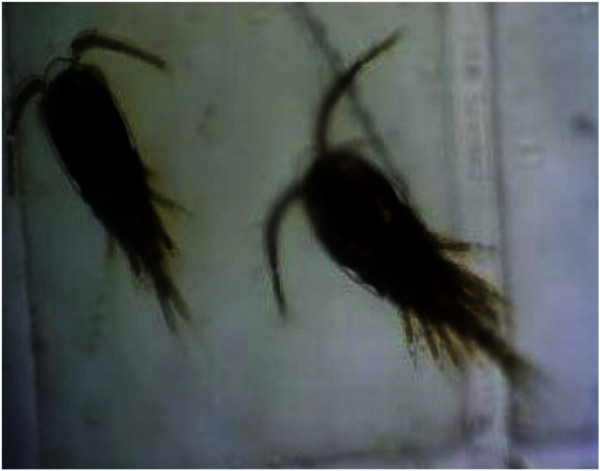
***Mesocyclops.***

Regression equations and calculated values (t) between zooplankton abundance and different water quality parameters that were found in semi-intensive culture systems are given by Table 5 (Figures 3,4,5,6,7 and 8).

**Table 5:** **Regression equations and calculated values (t) between zooplankton abundance and different water quality parameter**

Relationship	Regression equation	Calculated value (t)
Total Zooplankton Vs water temperature	Total zooplankton = 494.6 + 28.14*water temperature	.831
Total Zooplankton Vs Salinity	Total zooplankton = 1659 + (-)117.3*Salinity	−1.113
Total Zooplankton Vs pH	Total zooplankton = -28.959 + 171.87 *pH	.676
Total Zooplankton Vs Transparency	Total zooplankton = 2483.4 + -53.589*Transparency	−1.924
Total Zooplankton Vs DO	Total zooplankton = 117.3 + 19.822 *DO	.227
Total Zooplankton Vs Free CO2	Total zooplankton = 792.67 + 57.767* Free CO2	.672
Total Zooplankton Vs Hardness	Total zooplankton = 801.44 + .3821*Hardness	.878
Total Zooplankton Vs Alkalinity	Total zooplankton = 1088.1 + 1.1057*Alkalinity	.559

## Discussion

During the study period the zooplankton was dominated by copepods (54%). Islam *et al.*(2007) found, Copepoda as a dominant group in two shrimp’s gher at Khulna, Bangladesh. Similar result was observed by Ganapati (1943) and he found that copepod was a dominant order among zooplankton. Though Ali *et al.* (1985), Alam *et al.* (1987), Ali *et al.* (1989) and Mathias (1991) found Rotifera as a dominant group.

During the present study a distinct fluctuation of zooplankton population in different months (July to December, 2008) was observed. This fluctuation was the impact of different physico-chemical parameters on zooplankton population. Zooplankton population was observed similar in the area between the north-east coast of Australia and Indonesia by Harvey (1934). Similar observations were noted by Krisnomoorthi and Visvesvara (1966), Michel (1968), Mathew (1975), Ali *et al.* (1980), Chowdhury *et al.* (1987), Mathias (1991) and Islam *et al.* (2000) in different working areas. The study of Patra and Azadi (1987) in Halda River in Bangladesh showed similar plankton composition. The bulk of the zooplankton consisted of Rotifers, Cladocerans, Copepods, Crustacean and Insect Larvae.

During the present study a total of 11 genera of different group of zooplankton were identified from the study farm. The maximum amount of zooplankton was found at the month of October (20%) and minimum (14%) amount was found at the month of November. So, the maximum amount was found at the last summer season and minimum at early winter season. The zooplankton showed its peak in last summer. Such single peak was recorded by Miah *et al.* (1993) from a fish pond at Mymenshing. George (1964) observed maximum population of zooplankton in November, January and April to September and the major pulse was in June with 1399 units/L was observed. Patra and Azadi (1987) also found the peak in early winter from the Halda River in Bangladesh. But in the prawn farm which was semi-intensive maximum zooplankton was found at summer season because the farmers used artificial feed at maximum amount (body weight 8%) and the flow of rain water contained higher amount of nutrient. The similar result was found Islam *et al.* (2008), worked into two culturable ponds at southern part of Khulna.

Temperature is one of the most outstanding and biologically significant phenomena of aquatic environment; it has the relationship on zooplankton variation. In pond, the range of water temperature was found 22°C to 30°C. The maximum temperature was 30°C in August and minimum in December (22°C). The highest temperature during summer months was reported by Das and Bhuyan (1974), Islam *et al.* (1974) in Bangladesh. The low temperature was found in winter was supported by Das and Bhuyan (1974). The rainfall and air temperature has the direct influences on the variation of water temperature (Michael, 1968). Zooplankton abundance showed poorly positive correlation with water temperature in semi-intensive culture. In the pond Rotifera (r = -.805), Copepoda (r = +.668), Cladocera (r = -.096), Ostracoda (r = -.320), and such finding resembles the works of Chowdhury *et al.* (1987), Patra and Azadi (1987) and Islam *et al.* (2000). Ostracoda, crustacean larvae shows highly significant with water temperature.

Fluctuation of the limit of visibility is inversely related with turbidity. Transparency depends on zooplankton abundance and other organic particles. The range of transparency was 18 cm to 26 cm at the study period. The lowest transparency was found in July and the highest transparency was found in December. Zooplankton abundance showed slightly negative relationship with transparency in semi-intensive culture (r = -0.693). This findings support the results of Islam *et al.* (2008), they worked at two culturable ponds at southern part of Khulna.

Zooplankton abundance showed slightly positive relationship with Free CO_2_ in semi-intensive culture system (r = +0.319). This result, support the results of Alam *et al.* (1987), Patra and Azadi (1987) and Islam *et al.* (2000).

Zooplankton abundance showed slightly positive relationship with water pH in semi-intensive culture (r = +0.320). This similar result was found George 1964, Alam *et al.* (1987), Patra and Azadi (1987, Chowdhury *et al.* (Chowdhury and Mazumder 1987) and Islam *et al.* (2000). Zooplankton abundance showed slightly positive relationship with dissolved oxygen in semi-intensive culture system (r = +0.113). On the other hand zooplankton showed direct relationship with dissolved oxygen in such finding resembles the works of Miah *et al.* (1993) and Alam *et al.* (1987)*.* Zooplankton abundance showed slightly positive relationship with Alkalinity in semi-intensive culture system (r = + 0.269). These results have similarity with the findings of Miah *et al.* (1981) and Alam *et al.* (1987). Zooplankton abundance showed slightly positive relationship with Hardness in semi-intensive culture system (r = +0.402). These results have similarity with the findings of Miah *et al.* (1981) and Alam *et al.* (1987). Zooplankton abundance showed slightly negative relationship with water salinity in semi-intensive culture (r = -0.486). These results have similarity with the findings of Islam *et al.* (2008); they worked at two culturable ponds at southern part of Khulna. Understanding the role of plankton and its relation to water quality will help producers make critical management decisions. The knowledge of the findings will also help to determine the productivity of the pond where semi-intensive culture system is practiced and can make awareness among fish farmer which culture is feasible and what will be feeding strategies. However, in-depth studies covering all the months and more frequency of sampling would be necessary to make concluding remarks on aquatic ecology of this culture system. The findings of the present study would be helpful as baseline information for developing monitoring, management and conservation of ecosystem in semi-intensive farm in future.

## Conclusion

The plankton is considered to be the best index of the biological productivity and the nature of aquatic habitat. In semi intensive prawn culture farm the growth of the individuals not only depends on the supplementary feed but also on the production of plankton. So the presence of specific type of plankton is crucial for successful growth of prawn and other aquatic organisms. The physico-chemical parameters and the zooplankton abundance showed some interrelationships. These relationships are helpful to understand the seasonal and spatial variation of zooplankton population. From the present study it was found that the zooplankton abundance varied seasonally and it showed direct or indirect relationships with the physico-chemical parameters. In the study area a total of 11 genera of different group of zooplankton were identified from the study farm. Among the collected zooplankton, the order Copepoda was dominant with 4 genus followed by Rotifera (2 genus), Cladocera (2 genus) Ostracoda (2 genus) and shrimp larvae (1 genus) also found. Present study was such an attempt to know the taxonomy, abundance and periodicity of zooplankton. However, more studies are required to make a complete list of available zooplankton as well as their impact on water quality in the semi-intensive shrimp farms of southwest coastal region of Bangladesh.
